# Prediction for Future Yaw Rate Values of Vehicles Using Long Short-Term Memory Network

**DOI:** 10.3390/s23125670

**Published:** 2023-06-17

**Authors:** János Kontos, Balázs Kránicz, Ágnes Vathy-Fogarassy

**Affiliations:** 1 Continental Automotive Hungary Ltd., 8200 Veszprém, Hungary; kontosjan@gmail.com; 2 Department of Computer Science and Systems Technology, University of Pannonia, 8200 Veszprém, Hungary; 3 Faculty of Information Technology, University of Pannonia, 8200 Veszprém, Hungary; balazskranicz@gmail.com

**Keywords:** vehicle dynamics, yaw rate, LSTM, neural network, experimental data

## Abstract

Currently, electric mobility and autonomous vehicles are of top priority from safety, environmental and economic points of view. In the automotive industry, monitoring and processing accurate and plausible sensor signals is a crucial safety-critical task. The vehicle’s yaw rate is one of the most important state descriptors of vehicle dynamics, and its prediction can significantly contribute to choosing the correct intervention strategy. In this article, a Long Short-Term Memory network-based neural network model is proposed for predicting the future values of the yaw rate. The training, validating and testing of the neural network was conducted based on experimental data gathered from three different driving scenarios. The proposed model can predict the yaw rate value in 0.2 s in the future with high accuracy, using sensor signals of the vehicle from the last 0.3 s in the past. The $R^{2}$ values of the proposed network range between 0.8938 and 0.9719 in the different scenarios, and in a mixed driving scenario, it is 0.9624.

## 1. Introduction

The automotive industry is a complex and dynamic sector that plays a critical role in the global economy. Car manufacturers have to meet stringent safety standards and regulations to ensure that the vehicles they produce are safe to operate and protect the occupants in the event of an accident. One of the main safety standards is ISO 26262 [1], which describes the handling of safety-related work products for the whole life cycle of the vehicle.

Currently, the number of conform and safety-critical functions in vehicles is drastically increasing. From the anti-lock braking system (ABS) to the advanced, almost self-driving functions such as adaptive cruise control (ACC), a wide range of functions work simultaneously together. The electronic stability control (ESC) is part of every newly sold vehicle. The ESC is responsible for keeping the vehicle on track in under- or oversteering situations. One of the main inputs of the ESC is the vehicle’s yaw rate. The yaw rate is the angular velocity around the center of gravity of the vehicle. The ESC calculates an error based on the difference between the measured and calculated yaw rate. The measured yaw rate comes from the vehicle’s integrated measurement unit (IMU), which contains accelerometers and gyroscopes for measuring the vehicle’s acceleration in different directions and the yaw rate. The predicted yaw rate is calculated using a vehicle model such as the single-track model [2]. Based on the difference between the measured and calculated yaw rate, interventions, such as brake pressure increase or decrease at least on one wheel, can be achieved.

During normal circumstances for moving the steering wheel or for pushing the accelerator or brake pedal, the vehicle will not react immediately. There is a short delay before the requested changes are realized on the vehicle level. During this period, the vehicle’s dynamic behavior is determined by the previous state of the pedals and steering wheel. The outcome of an ESC intervention cannot be seen in the sensor signals immediately but only after the delay mentioned. A model that can predict the future values of the yaw rate could eliminate this delay. With that model, it is possible to check the effect of different interventions based on the vehicle’s future stability.

In this study, an artificial neural network-based model was developed to predict the future values of the yaw rate. The developed model can predict the future values of the vehicle’s yaw rate with relatively low hardware demands. The novelty of the model developed is shown by the fact that the existing approaches from the literature can only predict the yaw rate’s current value and are unable to predict future values of the yaw rate. During our work, we aimed to use the output of the currently built-in sensors found in a newly bought vehicle as the input of the NN model. As a reliable prediction model shall be based on experimental data, three different driving scenarios were defined, and experimental data were collected for each in Veszprém and on Continental’s own test track nearby Veszprém. The collected data were preprocessed and separated into training, validating and testing sets for developing the neural network model. For forecasting the future values of the yaw rate, a Long Sort-Term Memory (LSTM) network was designed and fine-tuned. After the hyperparameter tuning of the developed network, the calculated and experimental data were also compared.

In summary, the main contributions of the article are: (1) A Long Short-Term Memory-based artificial neural network was designed and fine-tuned for predicting the future values of one of the key parameters in vehicle dynamics, the yaw rate. (2) The developed model can predict the yaw rate 200 milliseconds forward with high accuracy. (3) The study was based on three different driving scenarios containing experimental data. The predictive ability of the network was tested for the three driving scenarios and for a mixed driving scenario, and the results were compared along each driving scenario.

The rest of the article is structured as follows. Section 2 presents a review related to the literature on the most important vehicle parameter (mainly for yaw rate) prediction using different modeling techniques. In Section 3, the basic concepts and operation of the LSTM network, on which the model is based, are described (Section 3.1, Section 3.2 and Section 3.3), and then, it is demonstrated how this type of recurrent neural network was used to predict the future values of the yaw rate. In Section 3.4, the methodology for evaluating the network’s predicting capacity is described. In Section 4, the description of the real driving data collected for the research is given, including the description of the three driving scenarios. Section 5 contains the evaluation of the results of the research. This is followed in Section 6 by the evaluation and discussion of them. Finally, Section 7 provides a summary of the paper.

## 2. Literature Review

In this chapter, the most important articles from the literature are collected that are relevant for the prediction of the yaw rate and for the usage of artificial neural networks in safety-critical applications in vehicles.

The estimation of the state of the vehicle is a widely researched topic; many affected articles can be found in the literature [3,4]. The most researched topics are the vehicle’s sideslip angle and the vehicle’s velocity estimations. The vehicle’s sideslip is a crucial input for describing the basic behavior of the vehicle [5], and the vehicle’s body estimation plays a fundamental role in the dynamic control systems [6]. The roll and pitch rates are similar to the yaw rate but for another vehicle dimension. The roll rate refers to the rate at which a vehicle leans or rolls over during a turning action. The pitch rate refers to the rate at which a vehicle’s nose or tail ascends or descends during accelerating, braking or driving over bumps. Several studies can be found to predict these two important vehicle dynamic descriptors [7,8]. In addition, the roll rate has an important role not just in the automotive industry but in the shipping industry [9]. Artificial neural networks are widely used in the yaw rate control of the vehicle, but only as a control algorithm and not as an estimation method for the yaw rate [10,11]. The number of articles on yaw rate prediction is limited.

The available articles can be separated into two groups: in the first group, the proposed methods use a dynamic model-based solution, mainly based on filtering or an observer. In the other group, the methods use a data-driven estimator such as artificial neural networks.

The general drawback of the dynamic model-based solutions is the large amount of vehicle-dependent parameters. The recognition of these parameters is the bottleneck of spreading these methods. Novara et al. developed a filter design method (called Set Membership) for predicting the value of the yaw rate. The novelty of their approach is the introduction of a directly identified filter for the data without using an additional model [12]. Sum et al. introduced an estimation framework for four-wheel independently actuated autonomous vehicles. Their work used a novel tire model and an adaptive square-root cubature Kalman Filter estimation strategy. Their results were verified using simulated data [13]. Baffet et al. developed a two blocks method based on Extended Kalman Filter (EKF). The inputs are the currently available standard sensors. The first block contains an observer that calculates the tire-road forces. The observer in the second block estimates the longitudinal and lateral forces, sideslip angle, cornering stiffness and yaw rate [14]. Zhang et al. proposed an Extended H-infinity Kalman Filter (EH∞KF) to estimate the vehicle speed, yaw rate and sideslip angle. First, a three-degree-of-freedom model was established, and based on that, the EH∞KF estimator was used. In their work, simulation test results were used to develop and test the developed approach [15]. Shi et al. proposed a linear Kalman filter for the yaw rate estimation of vehicles. Additionally, magnetic angular rate and gravity (MARG) sensors were used to eliminate the magnetic disturbances of the yaw rate. The magnetometer was also used to determine the vehicle status. The correlation was also developed between the MARG and the gyroscope to check the vehicle’s state. The proposed algorithm was tested with experimental data [16].

Neural networks are highly adaptable and can learn from data without requiring a precise mathematical model of the system. They can automatically adjust their parameters during training to optimize performance. This flexibility is advantageous when dealing with dynamic or uncertain environments where the system dynamics may change over time. In contrast, Kalman filters rely on accurate system models and noise statistics. The conventional Kalman filter can be used for linear systems; however, the adaptive Kalman filter can also describe non-linear behavior [17]. They excel in scenarios where the system dynamics can be modeled accurately and the noise statistics are well-defined.

Several studies are about applying artificial neural network-based solutions in the safety-critical automotive system [18,19]. Among the artificial neural network-based solutions, the yaw rate is also an essential input for several vehicle state estimation methods [20,21]. A Long Short-Term Memory (LSTM)-based estimation method for predicting lateral acceleration was developed by Kong et al. The inputs of their solution are the four wheels’ speed sensors’ signals, the yaw rate, lateral and longitudinal acceleration, the longitudinal speed, the actual gear, throttle opening, engine speed and steering wheel angle. In their work, the LSTM neural network was used to predict the actual value of the lateral acceleration based on the input values from the last five timestamps. The main difference compared to the approach of this paper is that the authors of this article estimate only the current lateral acceleration value without providing any future prediction [22]. In contrast, in the current study, the aim was to predict the future values of the yaw rate of vehicles. Hermansdorfer et al. introduced a neural-network-based end-to-end vehicle modeling solution. The inputs of their solution were the longitudinal and lateral velocity, yaw rate, longitudinal acceleration, steering wheel angle, wheel torque for the rear left and right wheels and brake pressure for the front and rear axle. The network outputs were the longitudinal and later velocity, longitudinal and lateral acceleration and the yaw rate. In their work, one hidden layer was applied with 150 GRU (Gated Recurrent Unit) neurons. However, there is no future prediction available for the outputs mentioned [23].

The number of yaw rate recognition articles using computer vision is growing. The main advantage of this approach is that the yaw rate can be predicted without additional sensors using the already existing vehicle camera system. However, future predictions of the yaw rate can not be provided directly using this approach. Huang et al. developed a monocular camera-based deep learning framework called YAEN (yaw angle estimation network). Unfortunately, their approach requires multiple times more hardware resources than is available for an electronic brake system [24]. Cunliang et al. proposed a novel steering angle prediction YOLOv5-based end-to-end adaptive neural network control for vehicles. Their deep learning network was trained using real road images. The calculated steering wheel angle correlates well with the yaw of the vehicle. The drawback of this approach is the high hardware demand [25].

## 3. Yaw Rate Forecasting Using Long Short-Term Memory Neural Network

This section describes the theoretical background of the present research, the structure of the applied LSTM network, its computation methodology, its application in yaw rate forecasting and the evaluation methodology of the results.

### 3.1. Basic Concepts

The aim of this study was to develop a neural network model capable of predicting the future values of the vehicle’s yaw rate with high confidence. As the inputs of the neural network, the past and present values of the longitudinal and lateral acceleration ($a_{long}$, $a_{lat}$), the yaw rate (Φ), the steering wheel angle (δ) and the wheel speeds (*v*) were used. The longitudinal acceleration is the changing rate of the vehicle’s speed in a forward/backward direction. The lateral acceleration is perpendicular to the longitudinal acceleration. Both accelerations are measured in g. The yaw rate is measured in deg/s. The wheel speeds (FL—front left, FR—front right, RR—rear right, RL—rear left) are the most convenient vehicle parameters to describe its moving; they are measured in km/h. The steering wheel angle is the main control of the vehicle and is measured in deg.

**Figure 1:**
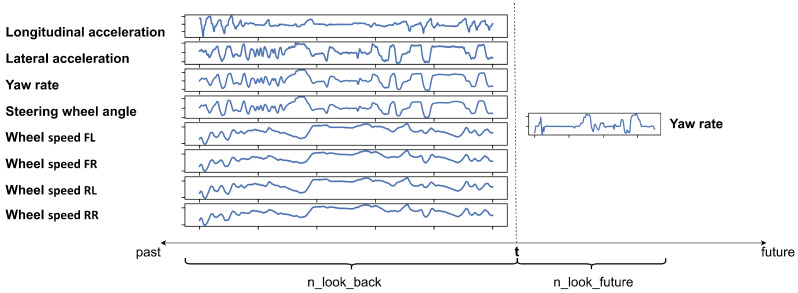
The concept of the developed model highlighting the input signals, the predicted output values and the meaning of the n_look_back and n_look_future parameters.

The network is expected to predict the future values of the vehicle’s yaw rate as an output. To achieve this goal, the LSTM neural network [26] was used; hence, a recurrent neural network can also capture the complex non-linear time-dependent dynamics of the vehicle. Since the LSTM network receives its input parameters from a time window and can predict future data projected to a time window, two parameters had to be introduced: the n_look_back and n_look_future parameters. The n_look_back defines how many consecutive historical data points preceding the current time should be considered for predicting the yaw rate’s future values. The n_look_future indicates how many future steps the neural network will predict. For better understanding, Figure 1 shows the meaning of the two parameters.

### 3.2. Introduction of the LSTM Cell

**Figure 2:**
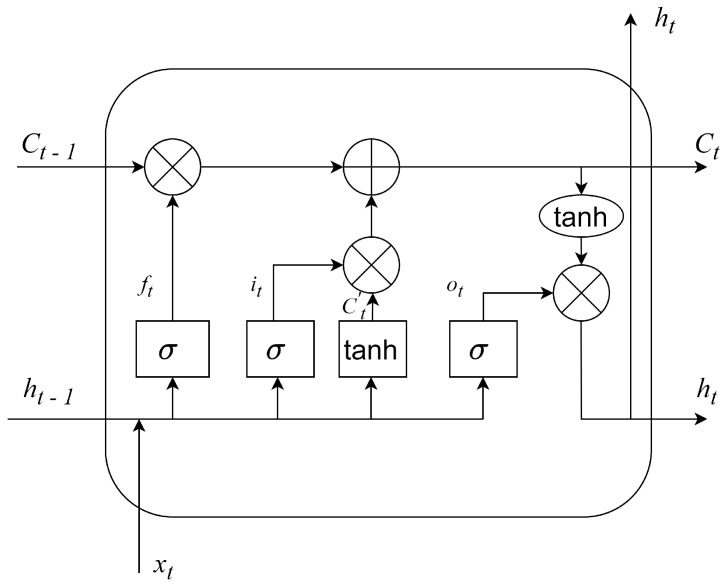
The structure of the used LSTM cell. Square notations containing σ and tanh indicate sigma and tanh-activated neural networks, and circles with ‘+’ and ‘×’ notations yield the pointwise vector addition (+) and multiplication (×).

The most important part of LSTM networks is the LSTM cell. The LSTM cell is a recurrent neural network unit that allows the model to remember information for extended periods. The basic structure of an LSTM cell consists of three main components: input gate (*i*), forget gate (*f*) and output gate (*o*). With these components, the LSTM cell combines the previous hidden state ($h_{t-1}$) (short-term memory of the network) with the new input ($x_{t}$) and updates the cell state (*C*), which represents the long-term memory of the network. The dimension of the hidden state ($h_{t-1}$) is [60, 1], which is the same as the number of outputs of the LSTM layer. The dimension of [$h_{t-1}$,$x_{t}$] is [120, 1]. The structure of the LSTM cell can be seen in Figure 2.

The forget gate decides which part of the cell state should be kept or forgotten for the current computation phase. It computes a weighted sum over the previous state and current input and applies a sigmoid activation function. This allows the model to forget information that is no longer relevant or useful. In our case, this means that the forget gate determines which historical information is no longer needed and/or is confusing for future yaw rate determination. The computation of the forget gate is given by Equation (1).

(1) $$f_{t}=\sigma \left(W_{f}\;\cdot \;\left[h_{t-1},x_{t}\right]+b_{f}\right)$$

where σ denotes the sigmoid activation function, and $W_{f}$ and $b_{f}$ are the weight matrix and the bias used.

The input gate determines which values from the current data will be used in the current iteration and helps control what information is added to the cell state. The computation of the input gate is described by Equations (2) and (3).

(2) $$i_{t}=\sigma \left(W_{i}\;\cdot \;\left[h_{t-1},x_{t}\right]+b_{i}\right)$$

(3) $$C_{t}^{\prime }=tanh\left(W_{C}\;\cdot \;\left[h_{t-1},x_{t}\right]+b_{C}\right)$$

where σ denotes the sigmoid and tanh the tanh activation functions, $W_{i}$ and $W_{C}$ are weight matrices, and $b_{i}$ and $b_{C}$ are biases. The $C^{\prime }$ is the new candidate cell state. The dimensions of $W_{f}$, $W_{i}$ and $W_{C}$ are equally [60, 90].

Following this, the cell memory (*C*) is updated at each iteration with information from the input and forget gates as follows:

(4) $$C_{t}=f_{t}*C_{t-1}+i_{t}*C_{t}^{\prime }$$

The output gate controls the new value of the hidden state ($h_{t}$), which goes back into the recurrent unit for the next time step. Thus, the output gate helps determine which parts of the short-term memory should be used at each step to produce meaningful results. The hidden states of the units in the last LSTM layer serve as the networks’ output. The computation of the output gate is given by Equations (5) and (6).

(5) $$o_{t}=\sigma \left(W_{o}\;\cdot \;\left[h_{t-1},x_{t}\right]+b_{o}\right)$$

(6) $$h_{t}=o_{t}*tanh\left(C_{t}\right)$$

where $W_{o}$ and $b_{o}$ are the applied weight matrix and the bias.

### 3.3. The General Structure of the LSTM Network Developed for Yaw Rate Forecasting

**Figure 3:**
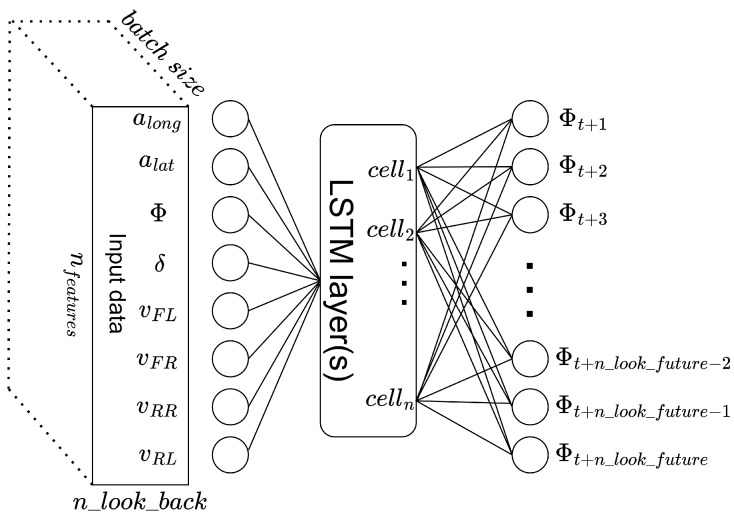
The structure of the developed LSTM network.

The general structure of the developed LSTM network consists of an input layer, some hidden layers and an output layer. The input layer transfers the input vectors to the network. The hidden layers consist of LSTM cells. The output layer provides the output to the environment. The network’s topology is shown in Figure 3.

The input layer of the network contains eight neurons, one for each type of input signal (past and present values of the yaw rate, lateral and longitudinal accelerations, steering wheel angle, four wheel speeds). The network receives the input as a three-dimensional data matrix, with the dimension of 8×n_look_back×batch_size. The size of the output layer is equal to the value of the n_look_future.

The tuning of the parameters and hyperparameters of the network was part of the study. In this phase, the aim was to find an optimal structure of the network that is capable of predicting the future yaw rate values with high accuracy, but the time complexity of the network remains low.

### 3.4. Evaluating the Predictive Capacity of the Network

For measuring the performance of the LSTM network, the following loss measures were calculated: mean squared error (MSE), root mean squared error (RMSE), mean absolute error (MAE) and $R^{2}$ score. The calculation of the error measures is given by Equations (7)–(10).

(7) $$MSE=\frac{1}{k}\sum_{i=1}^{k}{\left(y_{i}-\hat{y_{i}}\right)}^{2}$$

(8) $$RMSE=\sqrt{\frac{1}{k}\sum_{i=1}^{k}{\left(y_{i}-\hat{y_{i}}\right)}^{2}}$$

(9) $$MAE=\frac{1}{k}\sum_{i=1}^{k}\left|y_{i}-\hat{y_{i}}\right|$$

(10) $$R^{2}=1-\frac{\sum_{i=1}^{k}{\left(y_{i}-\hat{y_{i}}\right)}^{2}}{\sum_{i=1}^{k}{\left(y_{i}-\bar{y_{i}}\right)}^{2}}$$

where *k* denotes the number of samples, *y* is the expected value of the yaw rate, $\bar{y}$ is the mean value of *y*, and $\hat{y}$ is the yaw rate value predicted by the LSTM network. The expected values of the yaw rate were determined from the recorded datasets based on the values of the yaw rate sensor.

## 4. Datasets and Data Preparation

Training, validating and testing of the LSTM network were conducted based on experimental data. The data were collected by qualified test drivers on Continental’s official test track in Gyulafirátót, Hungary. A commercial SUV vehicle (produced in 2022) was used for the tests. For recording the data, an internal measurement system was used, which is capable of recording both the state of internal variables and the vehicle’s communication network. The collected data consisted of the vehicle’s longitudinal and lateral acceleration ($a_{long}$, $a_{lat}$), the yaw rate (Φ), wheel speeds of each wheel (*v*) and the steering wheel angle (δ). The sampling time was 10 ms. The recorded measurements were converted to CSV files with self-developed Python scripts.

For data collection, three different scenarios were defined: the *calm driver*, the *aggressive driver* and the *city driver*. The first two cases were recorded on the test track, while the last one was recorded in the city of Veszprém, Hungary. The average speed of the vehicle was between a standstill and around 50 km/h in all scenarios, and only the vehicle’s forward direction was considered. All three scenarios can be considered high-frictional tests. The measurements had no autonomous function intervention (e.g., ESC, ACC, ABS). In the *calm driver* driving scenario, the test pilot drove the car as if it happened on the city’s roads. During the *aggressive driver* maneuvers, the gradient of the deceleration/acceleration of the vehicle was higher than in the case of other scenarios. The total time of the collected data was 0.64, 1.67 and 1.96 h for the *calm*, *aggressive* and *city drivers*, respectively. The amount of *calm driver* related data was low compared to the other two scenarios, as this driving situation contained less diverse elements.

The data for each scenario were not recorded while driving continuously, but the test drivers were asked to stop for at least 5 s every 5–10 min. This made it possible to collect a significant amount of data on both stop and start events and to create a varied dataset from the data collected in the different driving situations. However, as the sensors were also operating during the standstill positions and these situations did not contain valuable information about the vehicle dynamics, these states were filtered out from all datasets. The basic condition for this filtering was that in standstill positions, the signals of the sensors were constant and the speed of all wheels was 0.11 km/h, which corresponds to the zero value of the wheel speed sensors. However, the states’ data, at which the steering wheel was moved during a standstill position, were kept, but the other sensors measured constant values (turning the steering wheel when the car is stationary). Following this data cleaning phase, data were present for 0.60, 1.55 and 1.82 h for the calm, aggressive and city scenarios, respectively.

The basic statistical evaluation of the data measured by the sensors in the three scenarios is shown in Table 1, Table 2 and Table 3.

The main differences between the driving scenarios are in the maximum and minimum values of the longitudinal and lateral accelerations, which means that in the case of the *aggressive driver* scenario, the forces that impact the car are higher. The same tendency can be discovered for the yaw rate: the maximum, minimum and quartile values (Q1, Q2, Q3 values) belonging to the yaw rate for the *aggressive driver* scenario are much higher than in the case of other scenarios. This means that the vehicle’s dynamics were much more intense in the *aggressive driver* driver scenario, which corresponds to the definition of an aggressive driving style. In the case of the *city driver*, the vehicle’s maximum speed value was a little bit lower than in the other two scenarios because in Hungary, 50 km/h is the official speed limit in urban areas.

The three databases were segmented into smaller sections due to the stops made at the request of the authors of this paper. Each section begins with a standstill and ends with a standstill state. Furthermore, 85% of each resulting fragmented database was selected randomly into the training set, while the remaining 15% was used as the test set. During the training, 12.75% of the training data was used for validation. Because in the study the n_look_back interval was selected to be large enough to contain all necessary information for the forecasting, the LSTM network was trained as a stateless network. In this way, the validation parts were always selected randomly.

## 5. Results

To find an optimal structure for the network, extensive parameter tuning was performed. During this activity, the aim was to design and implement a low-computational-complexity, yet high-accuracy neural network that can be built into an electric control unit with limited hardware resources. The hyperparameters of the neural network were tuned using a hybrid search method introduced in [27]. The maximum value of the n_look_future was set to 60, so that the developed network can predict future yaw rate values for a maximum of 600 ms. To find the best parameters and hyperparameters, the Adam optimizer [28] was applied with a learning rate of 0.001, and the monitored loss function was the mean squared error. To avoid overfitting, early stopping regularization was used with a patience value of 5. The research activity helped to find that a batch size equal to 512 was an optimal choice for training the network.

**Figure 4:**
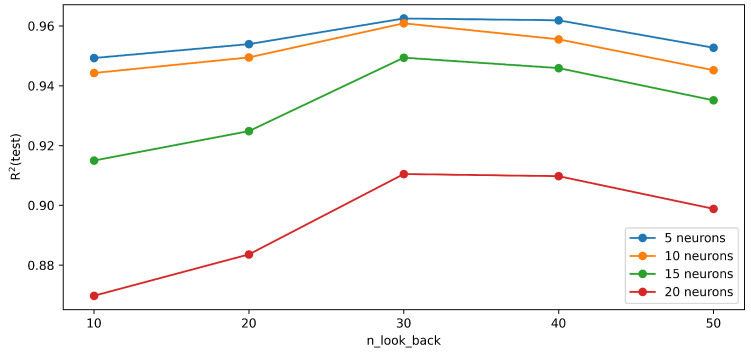
The value of $R^{2}$ in the case of the test data along the different n_look_back parameters.

The optimal value of n_look_back is a key point to obtain a good-performing model. In the case of n_look_back being too small, the necessary information for predicting the future value of the yaw rate is not included in the input data, which means that the amount of data is insufficient. On the other hand, in the case that the n_look_back value is too high, the computational time increases unnecessarily, and these excess data work as noise, which makes it more challenging to build a good-performing model. To find the best value for the n_look_back parameter, the performance of the LSTM network was investigated with different numbers of LSTM layers and different numbers of neurons per layer. Figure 4 shows the performance of the networks having different numbers of neurons in one LSTM layer as the function of n_look_back. The figure clearly shows that the optimal look-back interval into the past is 30 (300 ms), and the best performance is the network at which five neurons are placed into the LSTM layer. It was not only the performance of the network at the neuron counts shown in the figure that was evaluated, but all possible values below 10 were attempted. The best performance was obtained when the LSTM network had only one hidden layer and contained five neurons. The curve of the $R^{2}$ function was similar to that in the other cases, and the best n_look_back value was clearly 30.

**Figure 5:**
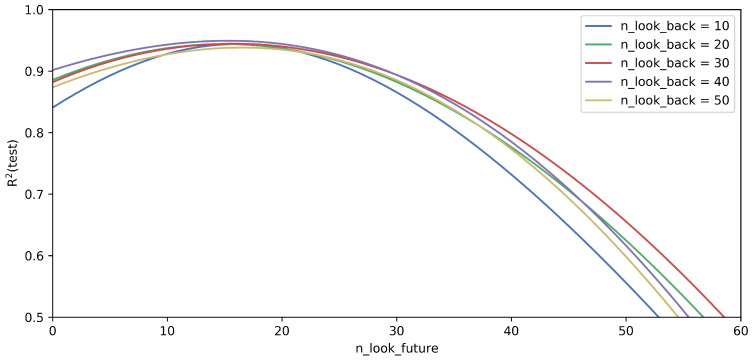
The value of $R^{2}$ in the case of the test data as a function of n_look_future. The investigated network contained one LSTM layer with five LSTM units.

Another interesting question is how the predictive ability of the network changes in the range of n_look_future as a function of time. To determine the optimal value of n_look_future, the developed model’s prediction power was examined for different n_look_back values with a fixed neural network configuration. Figure 5 shows the change in $R^{2}$ value as the function of n_look_forward for different n_look_back values in the case of the LSTM network with one hidden layer and five LSTM units. It can be seen that the best $R^{2}$ value belongs to n_look_future=20 (200 ms). In the case of a smaller n_look_future value, the vehicle’s behavior would also need data before the chosen n_look_back time interval. If n_look_future is higher than 20, the value of $R^{2}$ decreases significantly. The reason for this lies in the dynamics of the movement. To predict a yaw rate of more than 200 ms ahead, we would need data that will only be recorded by the vehicle’s sensors in the future. Comparing the curves with different n_look_back values, the previous observation is confirmed, i.e., n_look_back=30 is a good choice.

**Figure 6:**
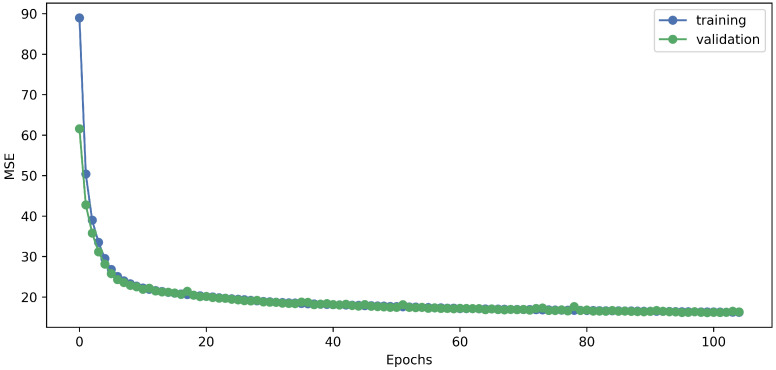
The training process of the developed LSTM network.

As a result of this research, it was found that the best-performing network had a single LSTM layer with five neurons and a look-back time window of 300 ms. The results of the parameter tuning are summarized in Table 4. The network with the resulting architecture has 640 parameters, ensuring high computational speed. With this architecture and parameters, the developed LSTM network’s training process showed a regular and smooth learning curve both for training and validation losses (Figure 6).

**Figure 7:**
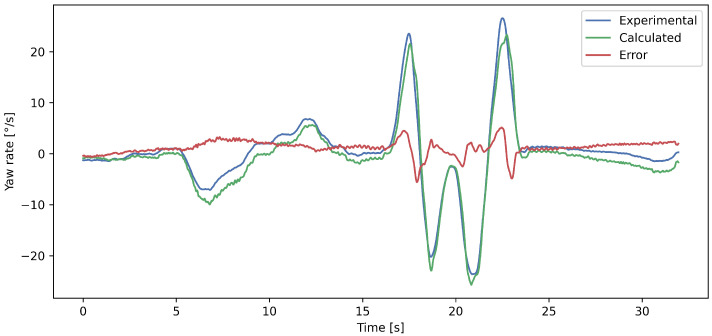
Comparison of the predicted and measured yaw rate values on the test set. The error is the signed difference between the experimental and calculated yaw rates.

**Figure 8:**
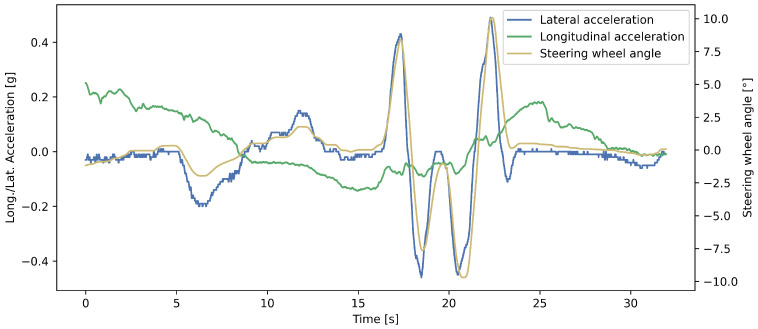
The longitudinal and lateral acceleration and the steering wheel angle from Figure 7.

Figure 7 shows the predicted and measured yaw rate values in a randomly selected time interval for visualizing the prediction performance of the developed network. It can be seen that the curves are close to each other. The developed model slightly underestimates the yaw rate value, however, not constantly. The steering wheel angle and the lateral and longitudinal acceleration from Figure 7 can be seen in Figure 8.

The numerical evaluation of the developed network’s performance for each dataset is given in Table 5. It can be seen that the best fits are for the training and validating sets, which are aligned with the expectation. It also shows that the homogeneity of the training and validating sets are similar. In the case of the testing set, the best results were gained for the *calm driver* ($R^{2}=0.9719$) and *aggressive driver* scenarios ($R^{2}=0.9706$). On the other hand, the result of the *city driver* ($R^{2}=0.8938$) is slightly lower compared to them. The main reason is that the diversity of the *City driver* scenario is limited compared to the other scenarios. In the general case, when the different scenarios were randomly mixed, the $R^{2}$ value of the network was 0.9624.

## 6. Discussion

The results of the study demonstrate that the developed LSTM-based neural network is capable of predicting the future values of the yaw rate for 0.2 s. The prediction horizon was chosen based on Figure 5 for 0.2 s. Besides the present study, other studies also showed a significant delay between the vehicle’s control and the outputs of the inertia sensors. This delay consists of the lag between the vehicle’s physical parts and the different sensors themselves [29]. Zhu et al. found that the delay depends on the vehicle’s speed and the driving scenario, but generally, it can vary between 50 and 200 ms [30]. In this study the delay was also 200 ms; thus, it is aligned with data from the literature.

The accuracy of the suggested model cannot directly be compared to other models from the literature because, according to the best of our knowledge, no model was published in the literature for predicting the future values of the yaw rate. Nevertheless, in this section, we try to compare the new results to those of similar models from the literature. Because of the lack of direct comparison, three articles estimating the yaw rate values in the current timestamp from sensor signals or other parameters were selected from the literature. These publications generally present the RMSE value as the error; thus, the comparison is also based on this error metric.

The EH∞KF filter proposed by Zhang et al. was fitted using 20 s of data [15]. In that study, the test maneuver was only an ISO-specified double-lane change. Furthermore, in that study, only simulated data were used. For these data, the RMSE value of the estimated and experimental yaw rate was given for 1.378 deg/s. Although the RMSE value is lower than in the case of the results of the present paper, this refers only to the actual values of the yaw rate and not to the next time moments. Furthermore, the disadvantage of the method is that it is a filter-based solution; thus, it requires several vehicle-dependent parameters as input, which could be complicated to recognize.

The artificial neural network-based model proposed by Hermansdorfer et al. was fit for three different driving scenarios, each containing a significant amount of data [23]. The RMSE value belonging to the current yaw rate value was 1.604 deg/s for their Monteblanco dataset. However, the application of the proposed network in an embedded system is complicated because of its high resource demand (150 GRU units in one layer). Although the authors published the used datasets, the wheel speed information was not included. Thus, a direct comparison between the models was impossible again. Moreover, similarly to the article mentioned previously, there are no future prediction data available for the yaw rate in that article.

In the article proposing the ‘Set Membership’ method, the authors used simulated and experimental data; however, the length of the experimental data was only 211 s [12]. For enlarging the data amount, 5000 data points of single-track model simulated data were also involved in the model development. Their M2 model provides the lowest RMSE value for a yaw rate of 1.089 deg/s. This model describes the yaw rate with the highest precision compared to the others. However, the amount of the used experimental data is limited, and no future prediction is available.

To summarize the facts, the RMSE error values, resulting from the model introduced in this paper, are remarkable when compared to other models, since the yaw rate values estimated here are not for the current time instant but are for the future. Knowing the future values of the yaw rate, it is possible to check the effect of different interventions, and the best one can be chosen from the point of view of safety.

The developed model’s usage is limited. It can only be applied to the same car type that was used for data collection. In addition, it is only valid for a speed range of 0–50 kph. For other car types or other speed ranges, the developed model shall be retrained with the newly collected data.

## 7. Conclusions

In this paper, an LSTM neural network-based solution was developed for predicting future values of the yaw rate. The model development was performed based on experimental measurements. The amount of collected and preprocessed data was almost 4 h, and they contain different driving situations. The data were collected on the test track of Continental in Gyulafirátót, Hungary, and in the city of Veszprém.

The proposed model is of low complexity, and it contains only one hidden layer with five LSTM neurons. For the prediction, the eight signal values of the vehicle (longitudinal and lateral acceleration, yaw rate, steering wheel angle and the four wheel speeds) were considered from the time interval of the last 300 ms.

The research concluded that the proposed model could predict the yaw rate values with high accuracy for 200 ms ahead. The MAE error of the model ranges between 1.067 and 2.201, and the RMSE error ranges between 1.932 and 2.754 for the different driving situations. The advantage of the model is its low computational demand, and a further advantage is that it does not require any vehicle-dependent parameter as input.

Based on our approach, it is possible to develop future prediction models for other descriptors of the vehicle. This level of safety in the case of autonomous and traditional vehicles could be increased.

## Figures and Tables

**Table 1:** Statistical properties of the *Calm driver*’s database.

	$a_{\mathrm{long}}$ (g)	$a_{\mathrm{lat}}$ (g)	Φ (^∘^/s)	δ (^∘^)	$v_{\mathrm{FL}}$ (km/h)	$v_{\mathrm{FR}}$ (km/h)	$v_{\mathrm{RR}}$ (km/h)	$v_{\mathrm{RL}}$ (km/h)
Mean	−0.011	0.010	1.992	0.717	30.83	30.97	30.81	30.67
Std	0.055	0.20	12.722	5.096	10.16	10.12	10.12	10.16
Min	−0.486	−0.68	−36.625	−28.861	0.11	0.11	0.11	0.11
Q1 (25%)	−0.039	−0.08	−4.186	−1.470	25.10	25.57	25.39	24.96
Q2 (50%)	−0.011	0.00	0.343	0.114	31.80	32.27	32.08	31.64
Q3 (75%)	0.015	0.13	9.664	3.398	38.01	37.97	37.84	37.87
Max	0.304	0.61	36.871	18.811	62.52	63.00	62.90	62.35

**Table 2:** Statistical properties of the *Aggressive driver*’s database.

	$a_{\mathrm{long}}$ (g)	$a_{\mathrm{lat}}$ (g)	Φ (^∘^/s)	δ (^∘^)	$v_{\mathrm{FL}}$ (km/h)	$v_{\mathrm{FR}}$ (km/h)	$v_{\mathrm{RR}}$ (km/h)	$v_{\mathrm{RL}}$ (km/h)
Mean	−0.013	0.00	1.096	0.351	36.28	36.36	36.15	36.06
Std	0.132	0.32	15.145	5.331	9.71	9.65	9.67	9.73
Min	−0.853	−1.03	−69.358	−33.477	0.11	0.11	0.11	0.11
Q1 (25%)	−0.064	−0.20	−8.145	−2.619	33.06	33.03	32.80	32.82
Q2 (50%)	−0.012	0.00	0.177	0.049	38.17	38.19	37.99	37.96
Q3 (75%)	0.054	0.22	10.090	3.133	42.57	42.56	42.39	42.39
Max	0.508	0.92	71.260	33.346	60.09	60.07	59.70	59.91

**Table 3:** Statistical properties of the *City driver*’s database.

	$a_{\mathrm{long}}$ (g)	$a_{\mathrm{lat}}$ (g)	Φ (^∘^/s)	δ (^∘^)	$v_{\mathrm{FL}}$ (km/h)	$v_{\mathrm{FR}}$ (km/h)	$v_{\mathrm{RR}}$ (km/h)	$v_{\mathrm{RL}}$ (km/h)
Mean	−0.005	0.01	0.025	−0.035	29.62	29.56	29.45	29.51
Std	0.062	0.07	5.820	4.080	13.14	13.12	13.12	13.14
Min	−0.372	−0.46	−35.513	−31.111	0.11	0.11	0.11	0.11
Q1 (25%)	−0.024	−0.01	−0.812	−0.363	20.51	20.68	20.56	20.40
Q2 (50%)	−0.009	0.00	−0.029	−0.021	32.53	32.46	32.38	32.42
Q3 (75%)	0.024	0.02	0.549	0.191	40.04	39.96	39.87	39.94
Max	0.309	0.46	35.255	33.187	56.40	56.26	56.63	56.67

**Table 4:** LSTM network structure obtained after hyperparameter tuning.

Parameters	Values
Number of input neurons	8
Number of hidden LSTM layers	1
- Number of neurons	5
- Transfer function	tanh
Number of output neurons	60
- Transfer function	linear

**Table 5:** Different error metrics for the measured and calculated yaw rate. Calm refers to the *calm driver*, Aggr to the *aggressive driver*, and City to the *city driver*. The column *All* contains the error values of a mixed scenario, where the data of the three driving scenarios were mixed randomly.

	*All*	Calm	Aggr	City
Training set				
Length (min)	166.5	25.0	64.9	76.6
$R^{2}$ (1)	0.9904	0.9957	0.9905	0.9814
MAE (deg/s)	0.678	0.577	0.936	0.492
MSE ((deg/s)${}^{2})$	1.223	0.682	2.136	0.626
RMSE (deg/s)	1.059	0.826	1.462	0.791
Validating set				
Length (min)	35.8	5.4	14.0	16.4
$R^{2}$ (1)	0.9906	0.9956	0.9907	0.9817
MAE (deg/s)	0.678	0.580	0.936	0.491
MSE ((deg/s)${}^{2}$)	1.204	0.695	2.106	0.604
RMSE (deg/s)	1.097	0.834	1.451	0.777
Testing set				
Length (min)	35.7	5.3	14.0	16.4
$R^{2}$ (1)	0.9624	0.9719	0.9706	0.8938
MAE (deg/s)	1.610	1.718	2.201	1.067
MSE ((deg/s)${}^{2}$)	5.404	4.791	7.583	3.734
RMSE (deg/s)	2.324	2.189	2.754	1.932
